# Application of computational approaches to study signalling networks of nuclear and Tyrosine kinase receptors

**DOI:** 10.1186/1745-6150-5-58

**Published:** 2010-10-11

**Authors:** Mouna Choura, Ahmed Rebaï

**Affiliations:** 1 Molecular and Cellular Diagnosis Processes, Centre of Biotechnology of Sfax, Route Sidi Mansour, Sfax, Tunisia

## Abstract

**Background:**

Nuclear receptors (NRs) and Receptor tyrosine kinases (RTKs) are essential proteins in many cellular processes and sequence variations in their genes have been reported to be involved in many diseases including cancer. Although crosstalk between RTK and NR signalling and their contribution to the development of endocrine regulated cancers have been areas of intense investigation, the direct coupling of their signalling pathways remains elusive. In our understanding of the role and function of nuclear receptors on the cell membrane the interactions between nuclear receptors and tyrosine kinase receptors deserve further attention.

**Results:**

We constructed a human signalling network containing nuclear receptors and tyrosine kinase receptors that identified a network topology involving eleven highly connected hubs.

We further developed an integrated knowledge database, denominated NR-RTK database dedicated to human RTKs and NRs and their vertebrate orthologs and their interactions. These interactions were inferred using computational tools and those supported by literature evidence are indicated. NR-RTK database contains links to other relevant resources and includes data on receptor ligands. It aims to provide a comprehensive interaction map that identifies complex dynamics and potential crosstalk involved.

Availability: NR-RTK database is accessible at http://www.bioinfo-cbs.org/NR-RTK/

**Conclusions:**

We infer that the NR-RTK interaction network is scale-free topology. We also uncovered the key receptors mediating the signal transduction between these two types of receptors. Furthermore, NR-RTK database is expected to be useful for researchers working on various aspects of the molecular basis of signal transduction by RTKs and NRs.

**Reviewers:**

This article was reviewed by Professor Paul Harrison (nominated by Dr. Mark Gerstein), Dr. Arcady Mushegian and Dr. Anthony Almudevar.

## Background

The receptor-ligand binding triggers a series of reactions in a living system. So, detailed knowledge and data about receptors such as nuclear receptors and receptor tyrosine kinases and their ligands are important for understanding living systems and diseases, and for designing new drugs.

Nuclear receptors (NRs) are one of the most abundant classes of transcriptional regulators in animals. They regulate diverse functions, such as homeostasis, reproduction, development, or metabolism. They are prominent pharmaceutical targets for diseases such as hypertension, cancer, diabetes, cardiovascular disease and the metabolic syndrome [1,2]. Nuclear receptors function as ligand activated transcription factors, thus providing a direct link between signalling molecules that control these processes and transcriptional responses. They bind to DNA as monomers, homodimers or heterodimers. The homodimers and heterodimers can bind to DNA elements that are oriented as palindromes, direct repeats, or even inverted repeats. The two dimerisation domains, that is the DNA binding domain (DBD) and ligand binding domain (LBD) work in tandem to enable DNA binding. The ability of NRs to bind different oriented repeats increases the level of complexity. The dimerisation network for human NR family revealed very connected hub-based topology [3].

Receptor tyrosine kinases (RTKs) transmit their activation signal across the plasma membrane, and many studies have demonstrated that the receptors, and not the growth factors, mediate the pleiotropic cellular responses. Growth factors recognize and activate their cognate receptors and stimulate receptor dimerization, tyrosine kinase activation and autophosphorylation. The autophosphorylated RTKs recruit and activate a receptor-specific complement of intracellular signalling pathways that relay information to the nucleus and other intracellular compartments [4].

Beyond this well-established mechanism of RTK and NR signallings, crosstalk between the RTK and NR receptors has been reported in many cases, such as

i) Complex interactions between steroid receptor (estrogen receptor (ER) and progesterone receptor) and growth factor receptor signalling governed breast cancer evolution [5].

ii) TrkB-GR interaction plays a critical role in the BDNF-stimulated PLC-γ pathway, which is required for glutamate release and the decrease in TrkB-GR interaction caused by chronic exposure to glucocorticoids results in the suppression of BDNF-mediated neurotransmitter release via a glutamate transporter [6].

iii) Nuclear RTKs regulate a variety of cellular functions, such as cell proliferation, DNA damage repair and signal transduction, both in normal tissues and in human cancer cells [7].

Here, our goal is to get a global understanding of the NR-RTK network using automated methods. We also developed a database which summarizes various data related to RTKs, NRs and their interactions and makes our results available for further discussion and investigation.

## Results and discussion

### NR-RTK signalling network

We integrated results derived from genomic context, high-throughput experiments, coexpression (conserved) and text mining related to human NRs and RTKs to obtain the complete dataset, with a total of 159 protein-protein interactions including 38 RTK-RTK, 42 NR-RTK and 79 NR-NR protein interactions as shown in Additional file 1.

**Figure 1:**
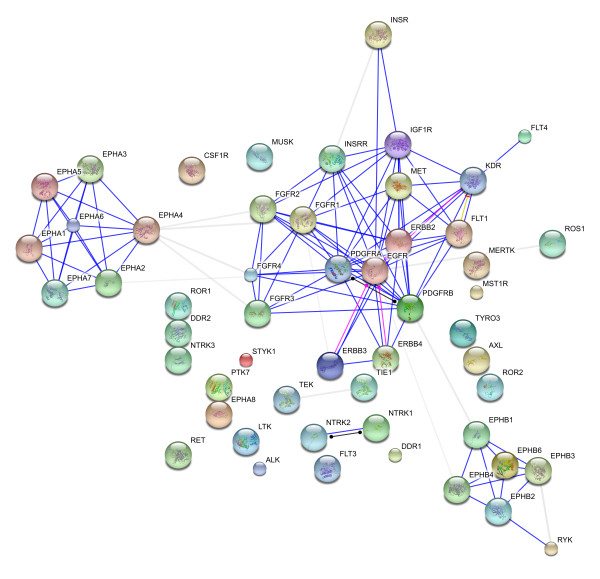
**RTK-RTK interaction network**.

**Figure 2:**
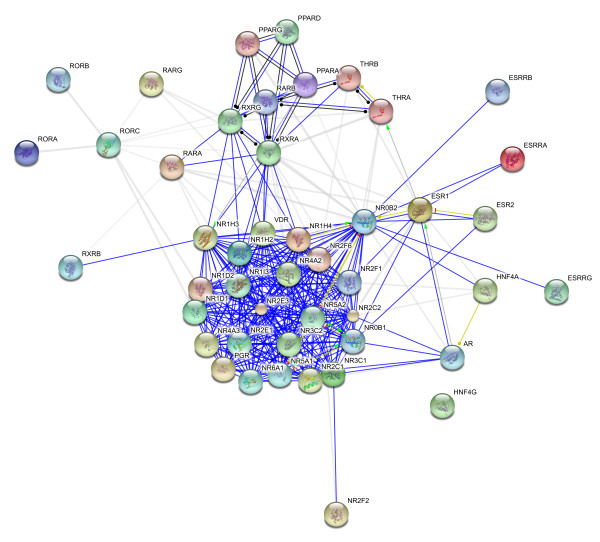
**NR-NR interaction network**.

We then analysed the RTK-RTK, NR-NR interaction networks separately. The RTK-RTK interaction network is shown in figure 1 revealing the central role of Erbb family, especially EGFR, in this very connected network (see additional file 1).To our knowledge, this paper presents for the first time the most thorough interaction dataset for the human RTKs. A similar observation for the very connected NR-NR interaction network (figure 2) showing the hub protein RXR [NR2B] (mentioned 14 folds as interactor) which is the common heterodimerising partner of 11 phylogenetic groups and SHP [NR0B2] (cited 15 fold as interactor) which is a co-repressor as mentioned in additional file 1. These results are in good agreement with the study of [3].

**Figure 3:**
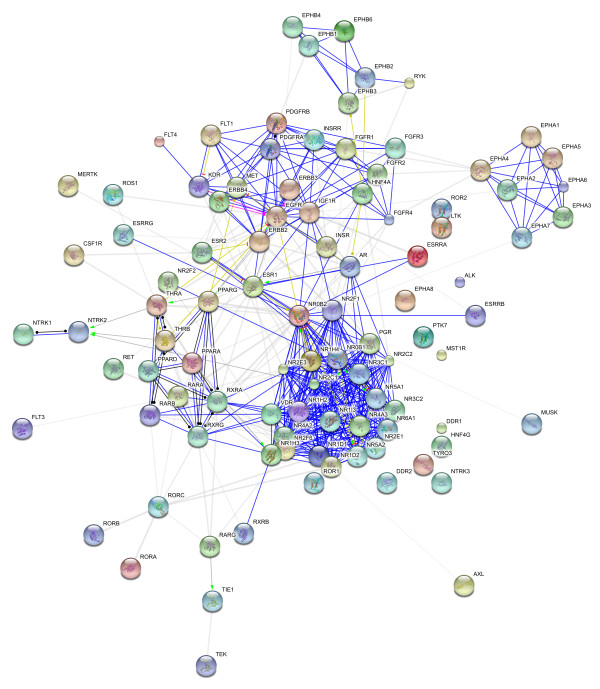
**NR-RTK interaction network**.

Our analysis reveals 42 NR-RTK interactions when the two networks were merged into one (figure 3, table 1, additional file 1). Moreover, we highlight the central role of Erbb family and steroid hormone receptors (EGFR, Erbb2, ESR1 and ESR2 are mentioned 7 times as interactors). This suggests that these connecting proteins are most likely responsible for propagation of transduction signal across the NR-RTK network.

**Table 1:** NR- RTK interactions and their combined scores

node1	node2	Combined score**
ALK	RARA	0.659
CSF1R	NR3C1	0.700
CSF1R	THRA	0.675
DHTR*	**ERBB2**	**0.786**
DHTR	EGFR	0.970
DHTR	ERBB3	0.478
EGFR	RARB	0.461
EGFR	THRA	0.820
**EGFR**	**ESR1**	**0.971**
EPHB4	NR3C1	0.467
EPHB6	ESR1	0.636
ERBB2	RARA	0.637
**ERBB2**	**ESR1**	**0.971**
ERBB2	THRA	0.810
ERBB2	NR1D1	0.441
ERBB3	ESR1	0.700
ERBB3	ESRRB	0.433
ERBB4	ESR1	0.550
ERBB4	PGR	0.588
ESR2	ERBB4	0.631
ESR2	ERBB2	0.777
ESR2	IGF1R	0.594
ESR2	ERBB3	0.689
ESR2	CSF1R	0.747
ESR2	MET	0.561
ESR2	EGFR	0.775
FGFR4	NR1H4	0.509
HNF4A	INSR	0.797
**IGF1R**	**ESR1**	**0.920**
NR2F2	PDGFRA	0.445
NR4A2	EPHB1	0.832
NTRK2	THRA	0.826
PDGFRB	NR3C1	0.545
PGR	EGFR	0.726
PGR	ERBB2	0.787
PGR	ERBB3	0.502
RARA	FLT3	0.666
RET	NR4A2	0.628
RET	RARA	0.402
RET	DHTR	0.478
THRA	ROS1	0.539

TIE1	NR2E1	0.682

* The interactions shown in bold are validated experimentally (see text for more details).

** Interaction score computed by STRING

Among the 42 predicted NR-RTK interactions, four interactions had been previously validated by *in vivo *or *in vitro *assays: DHTR-Erbb2 [8], EGFR-ESR1 [9], Erbb2-ESR1 [10] and IGF1R-ESR1 [11]. The interactions having high scores seem to be biologically relevant and worth to be validated experimentally (table 1). A higher confidence score may suggest a higher possibility of a putative protein-protein interaction (PPI).

On the other hand, we should take into account the substantial time lag between the first publication of a finding and at the time at which the PPI is entered in a database. It could therefore be postulated that many of our predicted PPIs predicted today are in fact correct, but have not been entered in a database for several years.

### Topology of the interaction networks

The topological properties of each network are listed in table 2. We tested the properties of the networks formed by the overall NR-RTK interactions, by plotting the log of frequency of proteins with *k *interactors against *log(k)*. For the whole NR-RTK and RTK-RTK networks, we obtained a log-log plot linear regression R^2 ^value of 0.794 and 0.899, respectively. This indicates that the networks have a scale-free topology. Nevertheless, this statistical property was not observed for the NR-NR protein interaction network (log-log plot linear regression R^2 ^= 0.39) (see additional file 1). Once again, this result is in good agreement with the study of Amoutzias [3] who inferred that human NR-NR interaction network is hub-based dimerization network in which a significant number of negative feedback loops is present, with the hub protein SHP [NR0B2] playing a major role.

**Table 2:** Network parameters calculated for each network by Network Analyzer

Parameters	NR-RTK	NR-NR	RTK-RTK
Number of nodes	86	45	35
Number of edges	1494	996	370
Clustering coefficient	0.57	0.732	0.653
Network density	0.142	0.351	0.19
Network heterogeneity	0.776	0.648	0.632
Network diameter	6	4	5
Network radius	3	2	1
Network centralization	0.312	0.466	0.36
Caracteristic path length	2.514	1.8	2.304

Average nb of neighbors	12.093	15.422	6.457

In scale free networks, a few nodes called hubs have higher degree than other nodes and play dominant role to conserve connections of the overall networks. Betweenness centrality is yet another global property of networks. High betweenness nodes occur on a large number of non- redundant shortest paths between other nodes. If a node is removed, it may disconnect different parts of the network. In order to identify important nodes involved in NR-RTK communication, we combined degree distribution and betweenness centrality measures. The highly ranked nodes are listed in Table 3 (all topological measures see additional file 2). Thus such nodes (NR0B2, EGFR, ESR1, NR2F1, ERBB2, RXRA, NR1D1, NR2F6, NR2C1, PGR and IGF1R) may be thought as potential bridges between NR and RTK networks and have most influence in the transmission of information (or cross talk) across the two networks.

**Table 3:** Potential hubs of NR-RTK network

Protein	Degree	Clustering coefficient	Betweenness
NR0B2	109	0.4	967.72
EGFR	78	0.25	998.81
NR2F1	83	0.63	929.03
ESR1	63	0.32	426.22
ERBB2	59	0.31	463.56
RXRA	69	0.41	386.69
NR1D1	64	0.77	330.41
NR2F6	81	0.71	322.12
NR2C1	76	0.75	268.92
PGR	73	0.73	216.71

IGF1R	53	0.41	208.98

### NR-RTK database content

NR-RTK database contains 624 protein receptors divided into 288 NRs and 336 RTKs curated from public databases. Among them 104 human protein receptors (48 NRs and 56 RTKs), the other vertebrate orthologs were taken from Chimpanzee, Dog, Mouse Rat and Chicken organisms.

The premise behind the design of the database was to utilize as many public, continually-updated databases as possible. Thus, the menu items are linked to resources such as the National Center for Biotechnology Information (NCBI), Pfam, MutDB, CGAP, PharmGKB (Table 4). Moreover, NR-RTK database includes information on the ligand (if it exists) of corresponding receptor.

**Table 4:** List of URL links in the NR-RTK database

Resources	URLs
NCBI	http://www.ncbi.nlm.nih.gov
PubMed	http://www.ncbi.nlm.nih.gov/sites/entrez?db=PubMed
Pfam	http://pfam.sanger.ac.uk/
MutDB	http://mutdb.org/
CGAP	http://cgap.nci.nih.gov/
PharmGKB	http://www.pharmgkb.org/

NR-RTK database is constructed through three modules. Each module performs specialized functions that provide users with a convenient way to find desired information according to the standard classification of these two kinds of receptors. The modules are described in the following sections:

### • RTKs module

Receptor tyrosine kinases are classified into 20 subfamilies according to their variable extracellular domain as described by [12].

### • NRs Module

Nuclear receptors are classified into seven groups (denoted from 0 to 6) according to Nuclear Receptor Nomenclature Committee.

### • NR-RTK interaction module

This module provides protein-protein associations derived from high-throughput experimental data, from the mining of databases and literature, and from predictions based on genomic context analysis.

This module contains data on NR-NR, RTK-RTK and NR-RTK interactions inferred as previously described. The data are collected in an Excel file containing interaction method detection, data mining references (PMID) and combined score for every association. From a functional perspective, 'association' can mean direct physical binding, but can also mean indirect interaction such as participation in the same metabolic pathway or cellular process.

NR-RTK database is accessible at http://www.bioinfo-cbs.org/NR-RTK/

## Conclusions

We infer that the NR-RTK interaction network is scale-free topology. We also uncovered the key receptors mediating the signal transduction between these two types of receptors. Furthermore, NR-RTK database is expected to be useful for researchers working on various aspects of the molecular basis of signal transduction by RTKs and NRs.

## Methods

### Datasets

We searched Swiss Prot Database for human NR and RTK and retrieved protein identifiers for 48 and 56 of them, respectively.

### Signalling network construction

Protein-Protein interaction network was constructed by STRING [13]. The interactions include direct (physical) and indirect (functional) associations; they are derived from four sources: genomic context, high-throughput experiments, coexpression and previous knowledge. These associations assigned a confidence score combining scores attributed to each source of evidence.

### Statistical properties and analysis of protein-protein interaction network

In order to assess whether a network is scale-free or not, the distribution of connectivity is plotted. Specifically, we plotted the log of frequency of proteins with K interactions, versus the log (K). A network may resemble a scale-free topology if the distribution of connectivity decays in a power-law fashion. Therefore, the better the trendline (in the log-log plot) fits a linear regression, the more the network resembles a scale-free topology [14].

The topological and statistical significance of network have been calculated using Cytoscape plugins Network Analyzer [15] and CentiScaPe [16].

The definitions of calculated parameters are available at http://med.bioinf.mpi-inf.mpg.de/netanalyzer/help/2.6.1/index.html.

### Database construction

All programming languages and software used were Open source, supplied under a general public license. We used the MySQL database server software http://www.mysql.com. The application runs on an Apache 2.0 HTML server http://www.apache.org.

## Abbreviations

DHTR: Dihydrotestosterone Receptor; EGFR: Epidermal Growth Factor Receptor; Erbb2: Receptor tyrosine-protein kinase erbB-2; ESR1: Estrogen Receptor 1; ESR2: Estrogen Receptor 2; IGF1R: Insulin-like growth factor 1 receptor; NR: Nuclear Receptor; NR0B2: SHP: Short heterodimer partner; NR1D1: nuclear receptor subfamily 1, group D, member 1; NR2C1: nuclear receptor subfamily 2, group C, member 1; NR2F1: nuclear receptor subfamily 2, group F, member 1; NR2F6: nuclear receptor subfamily 2, group F, member 6 PGR: Progesterone Receptor; RTK: Receptor Tyrosine Kinase; RXRA: Retinoic acid receptor RXR-alpha.

## Competing interests

The authors declare that they have no competing interests.

## Authors' contributions

MC did the computational analyses, data extraction and curation and database design. She also wrote the manuscript. AR supervised the work and corrected the manuscript. All authors read and approved the final manuscript.

## Reviewers' comments

### Reviewer's report 1

Paul Harrison Department of Biology, McGill University, Canada (nominated by Mark Gerstein, Biomedical Informatics, Yale University, USA

This paper describes a database of Nuclear Receptors and Receptor Tyrosine Kinases, and some analysis of their protein interactions.

* Firstly, it would be of benefit to researchers to make clear how much novel curation is involved in the construction of the database. At present, this is not entirely clear.

**Author's response: **We have added an explanation about the curation process in "NR-RTKdatabase content".

* Secondly, in the analysis of the interaction networks, the authors do not entertain other possible equation fits for the distribution of K_interactors versus Frequency (K_interactors). One should really check other possible equations also (not just a power law equation).

**Author's response: **We agree with the reviewer that we should check other equations. We feel that testing the scale-free topology and biological interpretation is sufficient for publication on this topic. As we are carrying on other works with the data, a more detailed comparison with other power law equation is desirable and we hope to complete that in the near future.

* Thirdly, in the database, the Pubmed links in the lists of families do not appear to work.

**Author's response: **Done, all links work.

* Fourthly, the authors should make sure that the format of the webpages and download files is adequately explained. Currently, there is not sufficient explanation. For example, there is not a detailed explanation of the columns in the Excel file of downloadable interactions. Help pages with this information should be provided.

**Author's response: **This information is explained in the manuscript in Results and Discussion section ("NR-RTK module"). Furthermore, we are now in the process of generating a new website to house these data.

### Reviewer's report 2

Arcady Mushegian, Department of Bioinformatics, Stowers Institute for Medical Research, Kansas City, Missouri, USA.

* This study is suitable for publication as a Discovery Note, not as a Research Article. Targeted reconstruction of a protein-centered interaction subnetwork by extracting and scoring all relationships of that protein may be of interest if this results in novel observations about biological system, and I would suggest to show more of it in this note.

**Author's response: **We don't think that this study can be considered as a Discovery Note. We believe that the significance of this work consists not just in the approach, but in the combination of methodology and biology to get new insights on the important receptors in signal transduction.

* For instance, instead of primarily focusing on the node degree of the network, the authors may discuss in more detail the actual gene content of the modules that they discover. How many of the connections and of module composition are well-known and how many are novel/not covered in the literature? Any new hypotheses suggested by the module inference? What type of evidence makes the most significant contribution to the network? Are there types of evidence that do/do not improve the score?

**Author's response: **The sub-networks discovered are RTK-RTK and NR-NR networks. The gene content of each sub-network is quite clearly described in the database such are their potential connections and their citations in literature. The module inference is exactly the main aim of the presented paper. Our approach may serve as predictive tool for indentifying key interactions and providing insight in experimental validation (*in vivo *and *in vitro *assays) (table1). The experimental assays and co-citations in literature for a given interaction improve the score.

The figures as they stand now are typical of many "systems-biology" papers, but it is not clear what to make of them. Are we supposed to eyeball the list of gene names (in which case the table would suffice)? Is the density of links supposed to be the main message? A position of particular nodes?

**Author's response: **The figures represent the interactions derived from different sources of evidence showing the central role of some proteins so called "hubs". The main point is the identification of these hubs according to their position in the network and other topological parameters (supplemental file 2).

Finally, about the "scale-free" character of the network. First, I am not sure that it is useful to compute the node degree distribution of the local, i.e., protein-centric network: if it is by construction a set of interactors of one protein, it is guaranteed that at least one protein will be very highly connected. More formally, it is known that fitting to the power law is not a right test here: many purported "scale-free" networks have been proven to reject the hypothesis in standard tests (e.g., Khanin and Wit, JCB 2006), or fit the power law only on an interval, or on different intervals with different values of the gamma parameter (discussed, for example, in several of Mark Newman's papers). Moreover, suppose that the distribution can be fit to a mixture of functions - what would the biological conclusion be?

**Author's response: **We agree with reviewer. Although the statistical test shows that the network is scale-free, we are also aware that much more tests should be performed especially for such complex system.

Regarding your question supposing a mixture of functions, this would be explained by two types of interactions: physical interactions and cross-talking with the two types of receptors that modulate gene expression.

**Reviewer's report 3: **Anthony Almudevar, Department of Biostatistics and Computational Biology University of Rochester Medical Center, Rochester, NY

The authors are concerned with two classes of proteins, nuclear receptors (NR) and receptor tyrosine kinases (RTK). These have broad functionality, with variants implicated in many disease states, including cancer. A database is constructed based on a protein-protein interaction (PPI) network of 48 NRs and 53 RTKs. The PPIs are compiled using various existing knowledge sources.

**Author's response: **We corrected the typing error in manuscript, 56 RTKs instead of 53 RTKs.

Three networks are analyzed, with NR proteins only (NR-NR), with RTK proteins only (RTK-RTK), and with all proteins (NR-RTK). It is reported that the RTK-RTK and NR-RTK networks have scale-free topology (ie. possess a power-law node degree distribution, with a small number of highly connected hubs, as is common in cellular networks). This does not hold for the NR-NR network, an observation which conforms to another published finding. A hypothesis for this observation is given, involving the presence of a large number of negative feedback loops.

Proteins which form highly connected hubs in the NR-RTK network (11 listed) are conjectured to influence the transmission of information between the NR and RTK networks.

The NR-RTK database is available at a URL given in the article. It is fairly basic in structure, but provides a useful way to explore the properties of the network and its components.

Overall, the paper is concerned with the construction of a knowledge database, and is not concerned with new methodology. As such, some potentially useful hypotheses concerning signaling pathways, and their role in disease states, are generated. The methods used seem sound. The given biological background, and hence the motivation for the database, is interesting.

Some suggestions:

(1) Abstract - Results: "We constructed a human signalling network ... that indentified a much more connected network topology than previously thought." Is it possible to provide citations, or to elaborate on this claim?

**Author's response: **To elaborate this result, we rephrased this sentence.

(2) In "Results and discussion" and "Methods: sections: The method used by the authors for testing the scale-free property is given in Barabási and Albert (1999) Science, vol 286, p -509-512. The exponent of the power law (the slope of the regression line) might also be reported, since it is sometimes used to characterize network properties, and might allow for a useful comparison to other cellular networks.

**Author's response: **We added this reference.

Minor points:

Background

- paragraph 4 - item iii): "of NR-RTK network" - > "of the NR-RTK network"

Results and discussion - NR-RTK signalling network

- paragraph 2: enclose "especially EGFR" in commas.

- paragraph 4: rephrase sentence starting "The remaining ..."

- paragraph 5: "and the PPI" - > "and the time at which the PPI ..."

Results and discussion - Topology of the interaction networks

- paragraph 2: "few nodes" - > "a few nodes"

- paragraph 2: "network" - > "networks"

- paragraph 2: "on large" - > "on a large"

**Author's response: **we corrected these points accordingly.

## Supplementary Material

Additional file 1 **RTK-NR interactions**. It contains in 4 worksheets 1) the protein-protein interactions, their scores, their source, the interaction detection method and PMIDs 2) NR-RTK distribution 3)RTK-RTK distribution 4) NR-NR distribution Click here for file

Additional file 2 **Topological coefficients of NR-RTK interaction network**. It contains in 6 worksheets 1) degree 2) Clustering coefficient 3)Closeness centrality 4)Eccentricity 5)Neighborhood connectivity 6) Average shortest path length. Click here for file
